# A functional polymorphism of microRNA-143 is associated with the risk of type 2 diabetes mellitus in the northern Chinese Han population

**DOI:** 10.3389/fendo.2022.994953

**Published:** 2022-09-23

**Authors:** Dexian Kong, Ya Duan, Jinli Wang, Yabin Liu

**Affiliations:** ^1^ Department of Endocrinology, Fourth Affiliated Hospital, Hebei Medical University, Shijiazhuang, China; ^2^ Department of Obstetrics, Hebei General Hospital, Shijiazhuang, China; ^3^ Department of Infirmary, Hebei Public Security Police Vocational College, Shijiazhuang, China; ^4^ Department of General Surgery, Fourth Affiliated Hospital, Hebei Medical University, Shijiazhuang, China

**Keywords:** MiRNA-143, polymorphism, type 2 diabetes mellitus, risk, expression

## Abstract

**Objective:**

To explore the association between two polymorphisms of microRNA-143 (miR-143) and the risk of type 2 diabetes mellitus (T2DM) in the northern Chinese Han population.

**Study Design:**

This case–control study involved 326 patients with T2DM and 342 healthy controls. Two genetic variants (rs4705342 and rs353292) of *miR-143* were genotyped by the polymerase chain reaction/ligase detection reaction (PCR-LDR) method. The levels of *miR-143* in the serum from 52 T2DM patients and 55 healthy subjects were investigated by quantitative real-time PCR (qRT–PCR).

**Results:**

The CC genotype frequency of rs4705342 was significantly higher in the T2DM patients than in the healthy controls (*P* = 0.012). After adjusting for sex, age, and body mass index, the rs4705342 CC genotype was also related to a significantly increased risk of T2DM compared with the TT genotype (adjusted OR: 1.87; 95% CI = 1.09-3.19; *P* = 0.022). Stratified analyses demonstrated that T2DM patients with the rs4705342 CC genotype had significantly higher levels of low-density lipoprotein cholesterol (LDL-C), fasting blood glucose (FBG), and glycated haemoglobin (HbA1C) than those carrying the rs4705342 TT genotype. The qRT–PCR results showed that the expression levels of miR-143 were significantly higher in the serum of cases than in the serum of controls (*P* < 0.001). Furthermore, the levels of miR-143 were significantly higher in the serum of T2DM patients carrying the rs4705342 CC genotype than in those carrying the TC and TT genotypes of rs4705342 (*P* = 0.005 and 0.003, respectively).

**Conclusion:**

The CC genotype of rs4705342 might be a risk factor for developing T2DM by increasing the expression of miRNA-143 in the northern Chinese Han population.

## Introduction

Type 2 diabetes mellitus (T2DM) is a complicated metabolic disorder leading to dysfunction in multiple organ systems. During the last three decades, the prevalence of this disease has increased dramatically worldwide, especially in developing countries (1, 2). In China, approximately 98.4 million adults (aged 20-79 years) were diagnosed with diabetes in 2013, and this number is expected to rise to 142.7 million by 2035 (3). Although the underlying mechanism of T2DM is not fully understood, it is now well established that the causes of this disease consist of complex interactions between genetic factors and environmental influences (4). To date, genome-wide association studies have identified numerous single-nucleotide polymorphisms associated with T2DM (5, 6).

MicroRNAs (miRNAs) are a class of small noncoding RNAs that participate in many biological processes, such as development, cell differentiation, proliferation, apoptosis and metabolic homeostasis. Recently, numerous studies have shown that dysregulation of miRNAs makes great contributions to the development of various diseases, including metabolic disorders (7–9). As a member of the miR-143/145 cluster, miR-143 functions in specifically controlling the metabolism of energy and lipids (10). miR-143 is reportedly involved in the differentiation of precursor adipocytes, and the expression levels of miR-143 are highly relevant to parameters of obesity and insulin resistance (11). Jordan et al. (12) demonstrated that upregulated miRNA-143 can damage the insulin-AKT signalling pathway and induce insulin resistance by silencing ORP8. In addition, miR-143 may also promote the development of insulin resistance by inhibiting autophagy (10). Insulin resistance is a major aetiological component for the onset of T2DM. Therefore, targeting miR-143 may be a potential therapeutic option for the prevention and treatment of T2DM.

To date, several genetic variants have been discovered in the miR-143/145 cluster. Among them, rs4705342 and rs353292 are two potential functional polymorphisms that might influence the expression level of miR-143 (13, 14). Previous studies have shown that these two polymorphisms are associated with the risk of chronic kidney disease, ischaemic stroke and colorectal cancer (15–17). However, little is known regarding the contribution of *miR-143* genetic variants to T2DM development. In this study, we explored the association between the two polymorphisms of *miR-143* and the risk of T2DM in the northern Chinese Han population. Furthermore, we analysed the levels of miR-143 in the serum of T2DM patients carrying different genotypes of the two polymorphisms.

## Material and methods

### Ethics statement

The study protocol was approved by the ethics committees of the Fourth Affiliated Hospital of Hebei Medical University, and all participants voluntarily signed a written informed consent according to the Declaration of Helsinki. This study was performed in conformity with the Strengthening the Reporting of Observational Studies in Epidemiology (STROBE) criteria.

### Study population

This case–control study involved 326 patients with T2DM and 342 healthy controls. All of the subjects were recruited consecutively between May 2018 and November 2021 from the Fourth Affiliated Hospital of Hebei Medical University. The diagnosis of T2DM was based on the World Health Organization diagnostic criteria with levels of fasting plasma glucose ≥ 7.0 mmol/L or 2-hour postprandial glucose ≥ 11.1 mmol/L (18). Basic data, including sex, age, body mass index (BMI), systolic blood pressure (SBP), and diastolic blood pressure (DBP), were collected from their medical charts. Patients were excluded if they had a history of other types of diabetes, autoimmune disorders, chronic hepatic or renal diseases, malignant tumours, and endocrine dysfunction. Controls were recruited from healthy volunteers who participated in the general health check-up at the Fourth Affiliated Hospital of Hebei Medical University. Exclusion criteria for control subjects were a personal or family history of diabetes mellitus, autoimmune diseases, cancer, or endocrine disorders.

### Biochemical analysis

Venous blood samples (6 mL) were collected from all participants after at least 12 h of fasting and divided into two portions: (1) one part was drawn into a vacutainer tube containing ethylenediaminetetraacetic acid for the assessment of glycated haemoglobin (HbA1C) levels and genetic analysis; (2) the other part was drawn into a plain vacutainer tube without anticoagulant to separate the serum by centrifugation for biochemical evaluation. In addition, a portion of serum from 52 T2DM patients and 55 healthy subjects was preserved at -80°C for miR-143 expression analysis.

The levels of fasting blood glucose (FBG), total cholesterol (TC), triglyceride (TG), high-density lipoprotein cholesterol (HDL-C), and low-density lipoprotein cholesterol (LDL-C) were measured using standard enzymatic methods on a Hitachi 7600-110 automatic biochemistry instrument (Hitachi, Japan). The levels of HbA1C were determined by the immunoturbidimetric technique.

### DNA extraction and genotyping

Genomic DNA was isolated from peripheral blood leukocytes using the salting-out method according to previously described by Miller et al. (19). The concentration and quality of the DNA was testeded by measuring the absorbance at 260 nm and 280 nm with a UV spectrophotometer (NanoDrop 2000; Thermo Scientific, Wilmington, DE, USA). The genotypes of rs4705342 and rs353292 polymorphisms were determined by the polymerase chain reaction/ligase detection reaction (PCR-LDR) method. The forward primer 5’- GGC TAG ATG CGG CAG ACC -3’ and the reverse primer 5’- CCA TGC CCC ACC TTT ATG C -3’ were used for PCR amplification of rs4705342 and rs353292. PCRs were conducted in a 15-μL mixture containing 1 μL of genomic DNA, 1.5 µL of 25 mmol/L MgCl_2_, 1.5 μL of 10 × PCR buffer, 0.3 μL of 10 mmol/L dNTPs, 1 U of Taq DNA polymerase (Tiangen Biotech), and 0.25 µL of forward and reverse primers (10 pmol/µL). The PCR cycling conditions were initial denaturing at 94°C for 2 minutes followed by 35 cycles of 94°C for 15 seconds, 55°C for 15 seconds, 72°C for 25 seconds, and a final extension at 72°C for 3 minutes. After the PCR fragments were subjected to LDR, the reaction products were analysed using an ABI 3730XL automated sequencer. Approximately 10% of the samples were selected at random to perform the repeated assays, and the results were 100% concordant.

### RNA isolation and quantitative real-time PCR

MiRNAs were isolated from the serum within six months after sampling using serum/plasma miRNA reagent (Beijing Solarbio Science & Technology Co., Ltd, Beijing, China) according to the manufacturer’s instructions. The concentration and quality of the RNA were evaluated using a UV spectrophotometer (NanoDrop 2000; Thermo Scientific, Wilmington, DE, USA). Total cDNA was synthesized by employing the ribo*SCRIPT*™ Reverse Transcription Kit (RiboBio Co., Ltd, Guangzhou, China). Quantitative real-time PCR (qRT–PCR) was conducted on a sequence detection system (ABI 7500; Applied Biosystems, USA) using miRNA Universal SYBR qPCR Master Mix (Vazyme Biotech Co., Ltd, Nanjing, China). The Bulge-Loop miRNA qRT–PCR Primer Set, including a specific stem–loop reverse-transcription primer and Bulge-Loop™ miRNA forward and reverse primers (RiboBio, China), was used for PCR. The sequences of the primers were not provided by the manufacturer. The relative expression level of miR-143 was calculated using the 2^-ΔΔCT^ method.

### Statistical analysis

The SPSS 22.0 statistical software package (SPSS Inc., Chicago, IL, USA) was used for all statistical analyses. A Hardy-Weinberg equilibrium analysis was conducted to compare the observed genotype frequencies of the controls with those expected using the chi-squared test. Clinical characteristics and laboratory data between the case and control groups were compared using the chi-squared test and Student’s t test. The chi-squared test was used to compare the genotype frequencies between the patients with T2DM and controls. Odds ratios (ORs) and 95% confidence intervals (CIs) were evaluated using unconditional multiple logistic regression models. One-way analysis of variance (ANOVA) was performed to compare the means of clinical and biochemical parameters among T2DM patients with different genotypes. The Mann–Whitney U test was applied to compare the miR-143 levels between cases and controls and among different genotypes. *P* < 0.05 was considered significant, and multiple comparisons were corrected by the Bonferroni correction.

## Results

### Clinical characteristics and laboratory data of the study participants

Baseline information of the study participants is presented in **Table 1**. The cases and controls were adequately matched in the distribution of sex (*P* = 0.844). However, the patients with T2DM had significantly higher age, BMI, SBP, DBP, TC, TG, FPG, and HbA1C than the controls (all *P* < 0.05). Genotype distributions of rs4705342 and rs353292 in the control group were consistent with Hardy-Weinberg equilibrium (*P* = 0.990 and 0.913, respectively).

**Table 1:** Clinical characteristics and laboratory data of study participants.

Variables	Patients (n = 326)	Controls (n = 342)	*P*
Sex (male/female)	155/171	160/182	0.844
Age (years)	61.99 ± 14.90	54.57 ± 17.93	< 0.001
BMI (kg/m^2^)	26.56 ± 2.91	23.78 ± 3.62	< 0.001
SBP (mmHg)	138.40 ± 22.04	127.98 ± 19.17	< 0.001
DBP (mmHg)	82.52 ± 14.38	80.42 ± 12.30	0.042
TC (mmol/L)	4.85 ± 1.22	4.60 ± 1.01	0.004
TG (mmol/L)	1.82 ± 1.12	1.44 ± 0.88	< 0.001
HDL-C (mmol/L)	1.30 ± 0.32	1.45 ± 0.33	< 0.001
LDL-C (mmol/L)	2.95 ± 0.91	2.76 ± 0.71	0.004
FPG (mmol/L)	8.30 ± 2.15	4.99 ± 0.65	< 0.001
HbA1C (%)	7.65 ± 1.66	5.52 ± 0.57	< 0.001

Data were expressed as mean ± standard deviation, and analysed by Student’s t test.

BMI, body mass index; SBP, systolic blood pressure; DBP, diastolic blood pressure; TC, total cholesterol; TG, triglycerides; HDL-C, high-density lipoprotein cholesterol; LDL-C, low-density lipoprotein cholesterol; FPG, fasting plasma glucose; HbA1C, glycated haemoglobin.

### Association of rs4705342 and rs353292 with the risk of T2DM

Genotype frequencies of rs4705342 and rs353292 in the case and control subjects are shown in **Table 2**. The CC genotype frequency of rs4705342 was significantly higher in the T2DM patients than in the healthy controls (*P* = 0.012). After adjusting for sex, age, and BMI, the rs4705342 CC genotype was also related to a significantly increased risk of T2DM compared with the TT genotype (adjusted OR: 1.87; 95% CI = 1.09-3.19; *P* = 0.022). However, no significant difference was observed between the case and control groups in the genotype distributions of the rs353292 polymorphism.

**Table 2:** Association between the two polymorphisms of miR-143 and the risk of T2DM.

Genotypes	Cases (%)	Controls (%)	OR (95% CI)	*P*	OR (95% CI) ^a^	*P* ^a^
	n = 326	n = 342				
rs4705342						
TT	128 (39.2)	160 (46.8)	Reference		Reference	
TC	146 (44.8)	147 (43.0)	1.24 (0.90-1.72)	0.194	1.29 (0.90-1.85)	0.167
CC	52 (16.0)	35 (10.2)	1.86 (1.14-3.02)	0.012	1.87 (1.09-3.19)	0.022
rs353292						
GG	253 (77.6)	271 (79.2)	Reference		Reference	
GA	64 (19.6)	66 (19.3)	1.04 (0.71-1.53)	0.846	1.07 (0.70-1.63)	0.761
AA	9 (2.8)	5 (1.5)	1.89 (0.63-5.72)	0.237	1.68 (0.50-5.61)	0.398

Data were expressed as a numbers (percentage), and analysed by chi-square test. P ^a^ value was obtained in multivariate logistic regression analysis.

T2DM, Type 2 diabetes mellitus; OR, odds ratio; CI, confidence interval.

a Adjusted for sex, age, and body mass index.

### Association of rs4705342 and rs353292 with clinical and biochemical parameters

The genotype distributions of rs4705342 and rs353292 in the T2DM patients, stratified by clinical and biochemical parameters, are listed in **Tables 3**, **4**. Compared with the rs4705342 TT genotype, the patients with the rs4705342 CC genotype had significantly higher levels of LDL-C, FPG, and HbA1C (**Table 3**). However, there was no significant association between rs4705342 and other parameters (**Table 3**). Stratified analyses also demonstrated that no significant association was seen between rs353292 and the clinical and biochemical indicators of T2DM patients (**Table 4**).

**Table 3:** Association between the rs4705342 genotypes and clinical characteristics of T2DM patients.

Group	Rs4705342 genotype			*P* value
	TT (n = 128)	TC (n = 146)	CC (n = 52)	
Male/female	67/61	62/84	26/26	0.244
Age (years)	62.82 ± 16.14	61.05 ± 14.16	62.60 ± 13.85	0.588
BMI (kg/m^2^)	26.61 ± 2.94	26.62 ± 2.90	26.24 ± 2.91	0.697
SBP (mmHg)	137.93 ± 22.38	138.53 ± 21.11	139.17 ± 24.11	0.939
DBP (mmHg)	81.30 ± 14.97	83.04 ± 13.42	84.08 ± 15.52	0.423
TC (mmol/L)	4.79 ± 1.25	4.87 ± 1.14	4.92 ± 1.39	0.774
TG (mmol/L)	1.81 ± 1.09	1.79 ± 1.01	1.90 ± 1.46	0.816
HDL-C (mmol/L)	1.30 ± 0.35	1.39 ± 0.30	1.31 ± 0.33	0.940
LDL-C (mmol/L)	2.79 ± 0.80	3.02 ± 0.88	3.17 ± 1.18	0.018
FPG (mmol/L)	7.85 ± 1.89	8.48 ± 2.37	8.94 ± 1.92	0.003
HbA1C (%)	7.37 ± 1.51	7.74 ± 1.75	8.08 ± 1.65	0.024

Data were expressed as mean ± standard deviation, and analysed by one-way ANOVA among genotypes. P values were corrected by the Bonferroni test.T2DM, Type 2 diabetes mellitus; BMI, body mass index; SBP, systolic blood pressure; DBP, diastolic blood pressure; TC, total cholesterol; TG, triglycerides; HDL-C, high-density lipoprotein cholesterol; LDL-C, low-density lipoprotein cholesterol; FPG, fasting plasma glucose; HbA1C, glycated haemoglobin.

**Table 4:** Association between the rs353292 genotypes and clinical characteristics of T2DM patients.

Group	Rs353292 genotype			*P* value
	GG (n = 253)	GA (n = 64)	AA (n = 9)	
Male/female	119/134	32/32	4/5	0.899
Age (years)	61.23 ± 15.36	64.75 ± 12.28	63.89 ± 18.01	0.223
BMI (kg/m^2^)	26.55 ± 2.89	26.66 ± 3.01	26.09 ± 2.99	0.856
SBP (mmHg)	138.87 ± 22.04	135.89 ± 22.10	142.89 ± 22.66	0.518
DBP (mmHg)	82.78 ± 14.42	81.91 ± 14.77	79.56 ± 11.06	0.748
TC (mmol/L)	4.88 ± 1.30	4.76 ± 0.91	4.79 ± 1.03	0.798
TG (mmol/L)	1.86 ± 1.19	1.66 ± 0.87	1.52 ± 1.65	0.311
HDL-C (mmol/L)	1.30 ± 0.32	1.29 ± 0.31	1.31 ± 0.51	0.992
LDL-C (mmol/L)	2.95 ± 0.96	2.99 ± 0.75	2.80 ± 0.76	0.842
FPG (mmol/L)	8.39 ± 2.19	8.06 ± 2.03	7.58 ± 1.88	0.332
HbA1C (%)	7.69 ± 1.68	7.59 ± 1.63	6.91 ± 0.76	0.366

Data were expressed as mean ± standard deviation, and analysed by one-way ANOVA among genotypes. P values were corrected by the Bonferroni test.T2DM, Type 2 diabetes mellitus; BMI, body mass index; SBP, systolic blood pressure; DBP, diastolic blood pressure; TC, total cholesterol; TG, triglycerides; HDL-C, high-density lipoprotein cholesterol; LDL-C, low-density lipoprotein cholesterol; FPG, fasting plasma glucose; HbA1C, glycated haemoglobin.

### Expression levels of miR-143 in the serum of T2DM patients and controls

**Figure 1:**
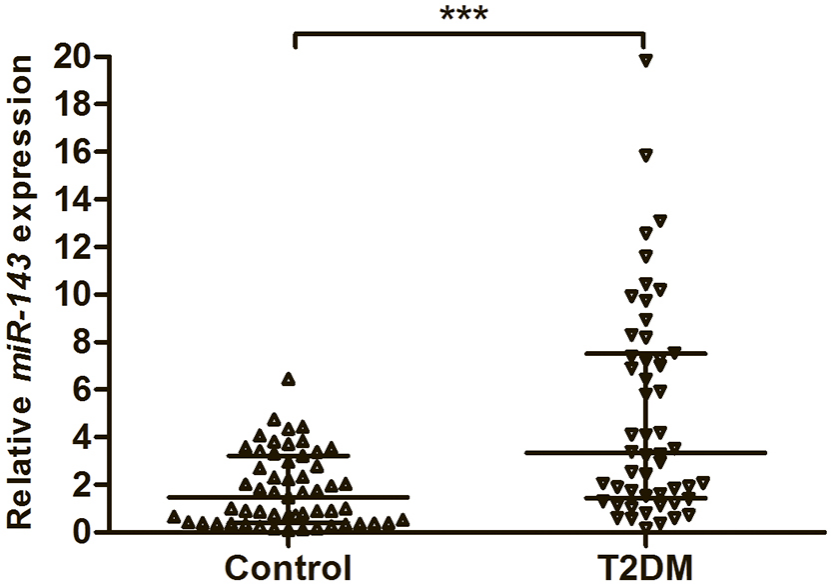
The expression levels of miR-143 in the serum from 52 patients with T2DM and 55 healthy subjects. Data were expressed as median with interquartile range, and analysed by Mann–Whitney U test. ***P < 0.001. T2DM, Type 2 diabetes mellitus.

To assess the expression of miR-143, we investigated 52 serum samples from T2DM patients and 55 serum samples from healthy subjects using qRT–PCR. The results showed that the expression levels of miR-143 were significantly higher in the serum of cases than in the serum of controls (*P* < 0.001) (**Figure 1**).

### Influence of rs353292 and rs4705342 on the expression of miR-143

**Figure 2:**
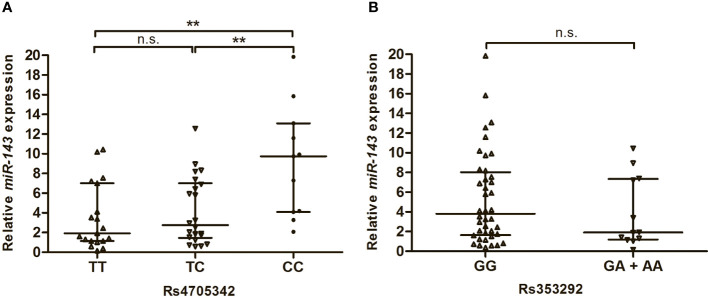
Impact of miR-143 polymorphisms on the miR-143 expression in the serum of Type 2 diabetes mellitus patients. Data were expressed as median with interquatile range, and analysed by Mann-Whitney U-test. **(A)** Relative expression levels of miR-143 in the serum from patients carrying the different genotypes of rs4705342. **(B)** Relative expression levels of miR-143 in the serum from patients carrying the different genotypes of rs353292. **P < 0.01. n.s., not significant.

In this study, we also analysed the expression levels of miR-143 in the serum of T2DM patients with different genotypes of rs4705342 and rs353292. As shown in **Figure 2**, the levels of miR-143 were significantly higher in the serum of T2DM patients carrying the rs4705342 CC genotype than in those carrying the TC and TT genotypes of rs4705342 (P = 0.005 and 0.003, respectively; **Figure 2A**). Nevertheless, there were no significant differences in miR-143 expression among the different genotypes of rs353292 (**Figure 2B**).

## Discussion

In the present study, we explored the impact of rs4705342 and rs353292 polymorphisms in *miR-143* on the risk of T2DM. The results demonstrated that the CC genotype of rs4705342 was significantly related to a higher risk of developing T2DM. Moreover, the expression levels of miR-143 were significantly increased in the serum of T2DM patients with the rs4705342 CC genotype compared with those with the TC and TT genotypes. To our knowledge, this study is the first to evaluate the association between genetic polymorphisms of *miR-143* and T2DM in a northern Chinese Han population.

Rs4705342 is a genetic variation located in the promoter of *miR-143*. Previous studies showed that the C allele of rs4705342 could reduce the protein-binding affinity and increase the promoter activity compared with the T allele (20, 21). Sima et al. (14) demonstrated that the rs4705342 CC genotype was associated with significantly increased miR-143 levels in the plasma of intracranial aneurysm patients. Similarly, we also found that the expression of miR-143 was significantly higher in the serum of T2DM patients with the rs4705342 CC genotype than in those with the TC and TT genotypes. These data further indicate that rs4705342 might be a functional genetic polymorphism. The C allele of rs4705342 has been reported as a risk factor for developing intracranial aneurysm and prostate cancer (14, 22). However, a protective role was observed for the rs4705342 C variant against cancer risk in a meta-analysis (23). In the present study, we assessed the association between the rs4705342 polymorphism and susceptibility to T2DM. Our results showed that the frequencies of the CC genotype of rs4705342 were significantly higher in the patients with T2DM than in the controls. This was consistent with the findings of Jahantigh et al. (24), who reported a significant correlation of the rs4705342 C variant with a higher risk of T2DM in a population from south-eastern Iran. In this study, we also found that the expression of miR-143 was significantly elevated in the serum of T2DM patients compared with healthy controls. Overexpression of miRNA-143 can cause insulin resistance and impair glucose metabolism (12). Therefore, we speculate that the CC genotype of rs4705342 may contribute to the development of T2DM by increasing the expression of miRNA-143.

Rs353292 is a common genetic polymorphism in the flanking region of miR-143. Yuan et al. (13) showed that the T allele of rs353292 exhibited a lower luciferase activity than the C allele, and the expression levels of miR-143 were significantly lower in colorectal cancer tissues from patients with the rs353292 TT/CT genotype than in those with the CC genotype. The relationship between this genetic variant and disease risk has been investigated extensively, but the results are controversial. The TT/CT genotypes of rs353292 were found to be associated with a higher risk of chronic kidney disease and colorectal cancer (15, 16). However, there was no significant correlation between the rs353292 polymorphism and susceptibility to papillary thyroid carcinoma and conotruncal heart defects (25, 26). In the present study, our results also demonstrated that the genotype frequencies of rs353292 were not significantly different between T2DM patients and controls. In addition, the expression levels of miR-143 were similar in the serum of T2DM patients carrying different genotypes of rs353292. These data indicated that the rs353292 polymorphism may have no effect on the susceptibility to T2DM in our study population. Of course, well-designed studies are needed to further elucidate the contribution of rs353292 to the risk of diseases and its influence on miRNA-143 expression.

A previous study suggested that miRNA-143 was associated with lipid metabolism (27). Takanabe et al. (11) showed that the expression of miR-143 was significantly upregulated in mesenteric fat from mice fed a high-fat diet, and increased miR-143 levels were related to an elevated body weight. In the present study, we also found that T2DM patients carrying the rs4705342 CC genotype had significantly higher levels of LDL-C than those carrying the rs4705342 TT genotype. Moreover, the miR-143 levels were significantly higher in the serum of T2DM patients with the rs4705342 CC genotype than in those with the TC and TT genotypes. Our results may be supported by the findings of Can et al. (28), who showed that the expression level of miRNA-143 exhibited a significant positive correlation with circulating LDL-C levels.

Some major limitations of this study should be mentioned. First, the sample size was relatively small, as the study included only 326 patients with T2DM and 342 controls. Second, all of the study subjects were unrelated individuals from a Chinese Han population recruited from the Fourth Affiliated Hospital of Hebei Medical University; thus, our findings cannot be directly applicable to other races or regional groups. Therefore, larger-scale and multiethnic studies are necessary to validate our results.

In conclusion, our study showed that rs4705342 may be a functional genetic polymorphism that affects the expression of miRNA-143. The CC genotype of rs4705342 might be a risk factor for the development of T2DM by increasing the expression of miRNA-143 in the northern Chinese Han population. Of course, these conclusions should be confirmed in a larger population-based study from different racial backgrounds.

## Data availability statement

The original contributions presented in the study are included in the article/Supplementary Material. Further inquiries can be directed to the corresponding author.

## Ethics statement

The studies involving human participants were reviewed and approved by the ethics committees of the Fourth Affiliated Hospital of Hebei Medical University. The patients/participants provided their written informed consent to participate in this study.

## Author contributions

YL: Project development, data collection, and manuscript writing. DK: Material preparation, data analysis and manuscript writing. YD: Data analysis and manuscript editing. JW: Data collection. All authors contributed to the article and approved the submitted version.

## Funding

The study was supported financially by a grant from the Science and Research planning of Health Committee of Hebei Province, China (No. 20180518), and the Natural Scientific Foundation of Hebei Province (Grant number: H2021206177).

## Acknowledgments

The authors would like to acknowledge the doctors in the Department of Endocrinology of the Fourth Affiliated Hospital of Hebei Medical University, China, for their assistance in recruiting study participants.

## Conflict of interest

The authors declare that the research was conducted in the absence of any commercial or financial relationships that could be construed as a potential conflict of interest.

## Publisher’s note

All claims expressed in this article are solely those of the authors and do not necessarily represent those of their affiliated organizations, or those of the publisher, the editors and the reviewers. Any product that may be evaluated in this article, or claim that may be made by its manufacturer, is not guaranteed or endorsed by the publisher.
